# The Impact of Qigong and Tai Chi Exercise on Drug Addiction: A Systematic Review and Meta-Analysis

**DOI:** 10.3389/fpsyt.2022.826187

**Published:** 2022-03-08

**Authors:** Jiabao Cui, Fang Liu, Xuan Liu, Ru Li, Xiaorong Chen, Hongfa Zeng

**Affiliations:** Faculty of Physical Education, Shenzhen University, Shenzhen, China

**Keywords:** mind-body exercise, addiction, craving, mental health, meta-analysis

## Abstract

**Background:**

Previous preliminary studies have found that qigong exercises produced significant effects in healthy people and in various clinical populations. The purpose of this study was to systematically review the effects of qigong and tai chi exercise on individuals with drug addiction.

**Methods:**

A systematic search of seven English databases and three Chinese databases was conducted to identify randomized controlled trials (RCTs) and non-randomized comparative studies (NRS) assessing the effects of qigong and tai chi on drug addiction. Study quality was assessed using the Checklist for the Evaluation of Non-Pharmaceutical Trial Reports (CLEAR-NPT).

**Results:**

Two RCTs and nine NRS studies were included in this study, including a total of 1072 patients with drug addiction (age range, 27–43 years). The results showed that qigong and tai chi exercise had a significant overall effect on depression (SMD = −0.353, 95%CI [−0.548, −0.159]), anxiety (SMD = −0.541, 95%CI [−0.818, −0.264]), quality of life (SMD = 0.673, 95%CI [0.438, 0.907]), and sleep quality (SMD = −0.373, 95%CI [−0.631, −0.116]). The subgroup analysis found that qigong outperformed tai chi on the improving depression, anxiety, and sleep quality.

**Conclusion:**

Existing studies suggest that qigong and tai chi are effective at improving depression, anxiety, and quality of life in drug users; however, the evidence from rigorous randomized controlled group trials is lacking.

## Introduction

The World Drug Report 2020 shows that ~35 million people worldwide are addicted to drugs (1). However, due to inadequate prevention and limited treatment resources, only 1 in 7 patients currently have access to treatment (1). Drug addiction is a chronic relapsing mental illness that afflicts millions of people (2, 3) and is closely related to the physical health of the patients. Drug addiction substantially increases the risk of suffering from various types of disease, including infectious illness, cancer, and chronic diseases (4, 5). As the number of detoxification sessions and length of drug use increase, the mental health of patients tends to decrease (6), and their chances of developing psychiatric disorders (e.g., major depression, bipolar disorder, specific phobias, antisocial personality disorder, and borderline personality disorder) increase substantially (7, 8).

In addition to the health problems, drug addiction causes loss of productivity, homelessness, and progressive violence, leading patients to be more prone to crime and other behaviors that endanger public safety (9–11). Currently, physiological detoxification methods (e.g., pharmacological interventions) are the main methods used by the international community to handle drug addiction (12–15). Although it is the most effective and direct method for controlling patients, the side effects are also very evident and lead to negative effects on patients' physical and mental health (16–19). Therefore, it is particularly important and urgent to find a safe and effective way to implement drug rehabilitation (20–22).

Previous studies have highlighted the benefits of exercise on the development of physical and mental health in healthy and clinical populations (23, 24). Some studies have suggested that exercise can be regarded as an adjunctive treatment for smoking cessation, alcoholism, and substance abuse (23–27). In addition to conventional forms of physical exercise, Qigong and tai chi have long been adopted for use by the National Institutes of Health as an alternative treatment of several chronic diseases (28, 29). Both Qigong and tai chi originated from ancient martial arts, which are based on four principles proposed by Larkey et al. (30). They include specific movement patterns or body postures, deep diaphragmatic breathing, a meditative state of mind, and a relaxed state of mind. There are several previous reviews showing that long-term qigong or tai chi exercise contributes to physical and mental health (22, 31–34). However, the underlying mechanism regarding the benefits of qigong and tai chi remains unconfirmed. One of the most accepted neurophysiological mechanism is that mindful concentration emphasized by qigong and tai chi exercise improves well-being by regulating the autonomic nervous system, specifically by upregulating the parasympathetic nervous system (35). Another assumption proposed that qigong and tai chi could benefit emotion regulation by mediating of the hypothalamic-pituitary-adrenal (HPA) axis (e.g., reduced saliva cortisol level) (35). One previous meta-analysis showed that moderate- to high-intensity mind-body exercises can be effective in increasing withdrawal rates from drug addiction, alleviating anxiety and depression, and serving as effective and sustained treatments for drug addiction disorders (22, 33, 34).

Qigong and tai chi have advantages over traditional methods of physiological detoxification because they are easy to learn and safe (36–40). Although qigong and tai chi detoxification have attracted the attention of researchers, most existing studies focused only on limited aspects, including the alleviation of depression (15, 41–44), anxiety (15, 41–43, 45), withdrawal symptoms (46), cocaine cravings (43), and improving quality of life (47–49), as well as sleep quality (41, 44). However, the effects remain unclear due to differences in study designs and the interventions performed (43, 50). Several previous reviews focus only on improvements in patients' anxiety and depression during drug withdrawal (43, 50), and limited to investigate the detoxification process, such as the correlation between patients' physical and mental health and the six indicators mentioned above, including withdrawal symptoms, quality of life, and cravings. Therefore, it is necessary to quantitatively analyze the effects of qigong and tai chi on drug addiction.

## Methods

### Literature Search

The literature search was conducted by two researchers, both of whom used an independent double-blind approach while searching the Medline (via PubMed), EMbase (via Ovid), PsychINFO (via Ovid), Eric (via EBSCOhost), SPORTDiscus (via EBSCOhost), CINAHL (via EBSCOhost), the Cochrane Central Register of Controlled Trials (CENTRAL), the China National Knowledge Infrastructure (CNKI), and Wanfang Data databases. In addition, a combined search for published papers related to the effects of qigong and tai chi on drug addiction was conducted on the Chinese Journal of Science (VIP) database from its date of inception to June 2020. This was conducted using Chinese search terms such as “fitness qigong,” “qigong,” “drug addiction,” “physical and mental rehabilitation,” “anxiety,” “depression.” A combined search was also conducted using English search terms such as “qigong,” “qi gong,” “heroin,” “morphine,” “recovery,” “treatment,” and so on. The search was limited to “human” as the subject, “English” and “Chinese article with an English abstract” as the language, and ‘peer-reviewed journal' as the literature type.

### Inclusion and Exclusion Criteria

#### Types of Studies

The included studies were randomized controlled trials (RCTs) or non-randomized comparative studies (NRS) published in peer-reviewed journals. Studies were considered RCTs if the study used and described a randomization method explicitly at the time of participant randomization (e.g., use of computerized random number generators, coin flips, or lotteries). Similarly, studies were considered NRSs if they were generated only by pseudo-random or non-random methods (e.g., use of date of birth, date of admission, participant preferences, or intervention usability). Studies were excluded if they were observational studies (e.g., cross-sectional, case control), reviews, conference abstracts, and book chapters that did not involve any comparisons of outcomes between groups.

#### Types of Participants

Participants included in the studies were patients aged 18 years and older with illicit drug abuse or dependence (based on DSM-3/4/5 as the diagnosis). Patients with drug abuse or dependence who also had alcohol or nicotine dependence or severe specific conditions (e.g., cancer, asthma) were excluded.

#### Types of Interventions

Studies in which the experimental group used any type of tai chi or qigong as an intervention and the control group used treatment as usual (TAU) or other types of interventions (e.g., medications) were included in the analysis. Studies in which the experimental group used tai chi or qigong with other interventions superimposed and studies in which static qigong (meditation or positive thinking) were excluded.

### Literature Screening and Data Extraction

**Figure 1:**
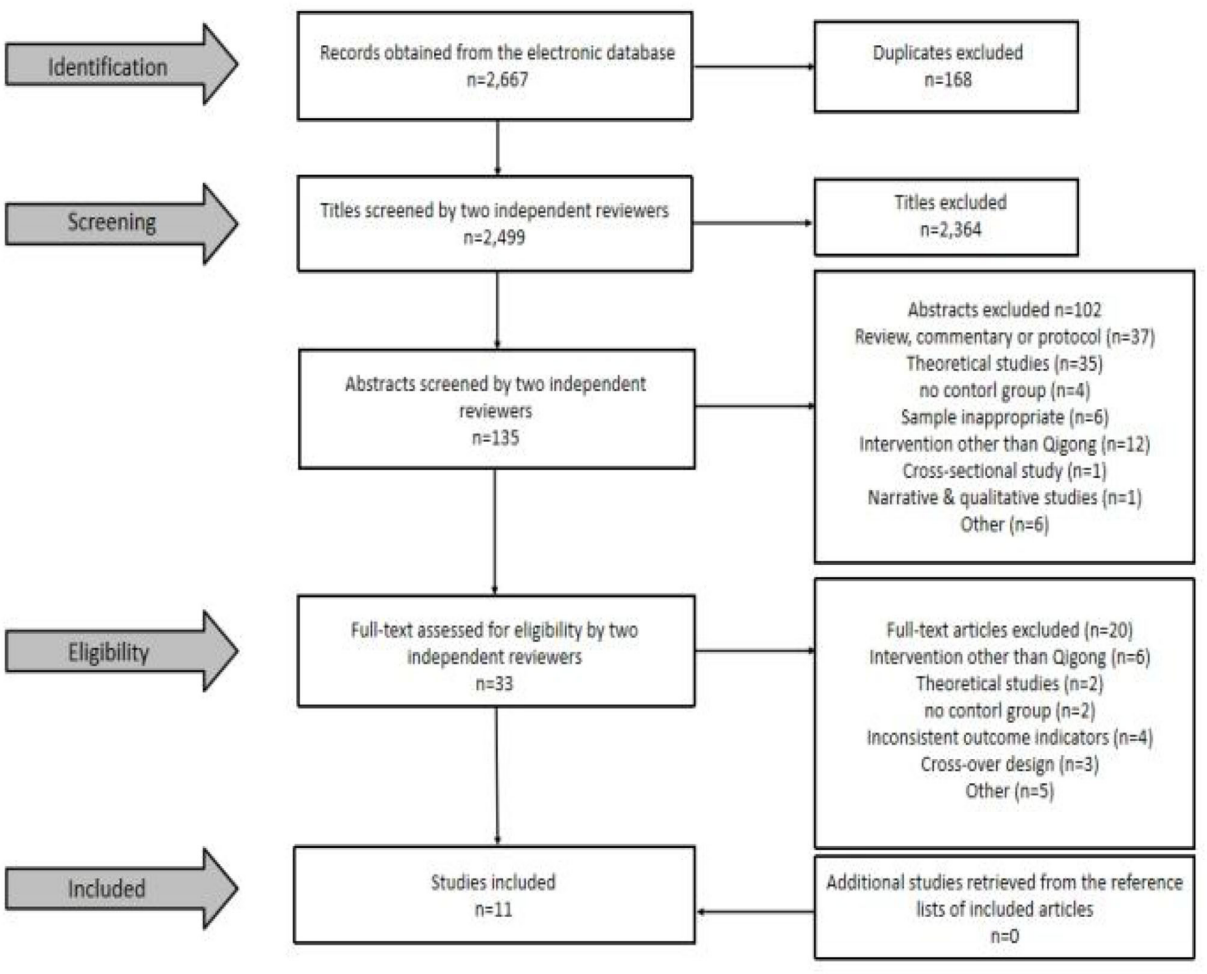
Selection process for included studies.

Literature screening was performed by two researchers (JC, FL) based on the inclusion and exclusion criteria. Judgment of exclusion was made by reading the titles and abstracts, and full-text downloading was performed after obtaining literature that largely met the inclusion criteria. Final judgment of inclusion was made after reading the full text in detail. If the opinions of the two researchers did not align, the final decision was made by a third researcher (RL) after a group consultation. Finally, one researcher (XL) looked for potentially missing articles from a review of the literature related to the effects of tai chi and qigong on addiction (Figure 1). The consistency of literature screening was tested by calculating the Kappa (K) coefficient, with K > 0.75 indicating good consistency, 0.4 ≤ K ≤ 0.75 indicating fair consistency, and K < 0.4 indicating poor consistency (51).

All relevant data were extracted from the screened literature, including the first author, year of publication, study population, sample size, gender, age, intervention period, intervention protocol, and outcome indicators. For literature with missing or unclear of data, we contacted the authors of the article to obtain any relevant information. If there was no response after three emails, we classified the study information as “Unsure” and rated it as “U” (Table 1).

**Table 1:** Characteristics of the included studies on the impact of qigong and tai chi exercise on drug addiction.

**References**	**Study design; location**	**Study participants**	**Sample size(Mean age ±SD)**	**Intervention**	**Control**	**Duration**	**Outcome measures**	**Results**
Li et al. (15)	NRS, Yunnan, China	Females with drug dependence on heroin	Exp: 36 (30.7 ± 6.3) Con: 34 (30.7 ± 6.3)	Qigong-Tai Chi (1 h, once every two days)	TAU	24 wk	HRSD	*p* = 0.049
Smelson et al. (43)	RCT, ??	Individuals with dependence on cocaine (sex unreported)	Exp: 45 (36.0 ± 9.4) Con: 41 (40.4 ± 11.9)	Qigong (15 min, 2–3 times per week, once every two days)	TAU	2 wk	(1) CCQ (2) BDI (3) STAI	(1) *p* = 0.06 (2) *p* < 0.05 (3) *p* > 0.05
Zhu et al. (44)	RCT, Shanghai, China	Females with dependence on amphetamine-type stimulant	Exp: 42 (33.74 ± 7.11) Con: 38 (37.76 ± 9.85)	Qigong-Tai Chi 24-form (1 h, five sessions per week for the first 3 months, and three times per week for next 3 months)	TAU	24 wk	(1) PSQI (2) SDS	(1) *p* = 0.027 (2) *p* > 0.05
Geng et al. (42)	NRS, Shanghai, China	Females with dependence on synthetic drugs	Exp: 30 (34.0 ± 7.0) Con: 30 (38.0 ± 5.0)	Qigong-Tai Chi 24-form (45 min, 5 times per week)	TAU	12 wk	(1) SCL-90 (Depression) (2) SCL-90 (Anxiety)	(1) *p* > 0.05 (2) *p* > 0.05
Fu et al. (41)	NRS, Anhui, China	Females with drug dependence on heroin, methamphetamine, k powder, and ecstasy	Exp: 100 (28.3 ± 7.83) Con: 100 (27.99 ± 8.17)	Qigong-Wu Qin Xi (30 min, once per day)	No treatment	20 wk	(1) SAS (2) SDS (3) PSQI	(1) *p* = 0.000 (2) *p* = 0.003 (3) *p* = 0.000
Huang et al. (45)	NRS, Zhuhai, China	Individuals with dependence on heroin (M = 68, F = 32)	Exp: 50 (35.26 ± 12.22) Con: 50 (35.21 ± 12.12)	Qigong-Ba Duan Jin (30 min, twice a day)	Medication	20 wk	SAS	*p* < 0.05
Li et al. (47)	NRS, Gansu, China	Individuals with dependence on opioid dependence (M = 160, F = 40)	Exp: 100 (41.47 ± 16.65) Con: 100 (40.18 ± 11.83)	Qigong-Dao Yin (1 h, once every days)	Regular physical exercise	12 wk	QOL-DA	*p* < 0.05
Zhu et al. (48)	NRS, Shanghai, China	Females with dependence on synthetic drugs	Exp: 43 (34.0 ± 7.0) Con: 39 (38.0 ± 10.0)	Qigong-Tai Chi (1 h, 5 times per week for the first 3 months, and 3 times per week for next 3 months)	TAU	24 wk	QOL-DA	*p* < 0.01
Zhu et al. (49)	NRS, Shanghai, China	Males with dependence on amphetamine-type stimulant	Exp: 30 (37.47 ± 8.41) Con: 29 (42.29 ± 11.37)	Qigong-Tai Chi 24-form (50 min, 5 times per week)	TAU	12 wk	QOL-DA	*p* = 0.002
Huang et al. (46)	NRS, Zhuhai, China	Individuals with dependence on heroin (M = 44, F = 16)	Exp: 30 (34.50 ± 4.90) Con: 30 (34.50 ± 4.60)	Qigong-Ba Duan Jin (30 min, twice every days)	Medication	24 wk	PWS	*p* < 0.05
Li et al. (55)	NRS Changzhou, China	Females with dependence on heroin	Exp: 34 (33.3 ± 6.5) Con: 26 (31.7 ± 6.1)	Qigong-Pan Gu (2–2.5 h, once every days)	TAU	1 wk + 3 d	HAMA	*p* < 0.01

*RCT, Randomized Controlled Trial; CCT, Controlled Clinical Trail; NRS, non-randomized comparison study; M, male; F, female; Exp, experiment group; Con, control group; TAU, treatment as usual; ??, not provided in the text; HRSD, Hamilton Rating Scale for Depression; CCQ, Cocaine Craving Questionnaire; BDI, Beck Depression Inventory; STAI, State-Trait Anxiety Inventory; SAS, Self—Rating Anxiety Scale; QOL-DA, Quality of life scale for drug addicts; SCL-90, Self-reporting Symptom Checklist 90; SDS, Self-Rating Depression Scale; PSQI, Pittsburg Sleep Quality Index; PWS, protracted withdrawal symptoms; HAMA, Hamilton Anxiety Scale*.

### Quality Assessment

The methodological quality of included studies were independently assessed by two researchers (JC, FL) based on the criteria of a Checklist to Evaluate a Report of a Non-pharmacological Trial (CLEAT-NPT) (52). The quality assessment followed criteria including randomization, concealed allocation, the availability of intervention details, appropriate experience of care providers, participant compliance, participant/caregiver blinding, blinding of outcome assessors, equality of follow-up schedules between groups, and intention-to-treat principles. The quality of the included studies was rated by calculating a quality rating index (0–100%) (53). The quality evaluation can be divided into two parts: [1] the quality of each study (cross-sectional) in terms of individual study units, based on the 10 criteria mentioned above, and [2] the quality of the included studies (as a whole) in terms of each individual criterion. Values below 60% were of substandard quality, those between 60 and 80% were considered to be of fair quality, and those above 80% were considered to be of good quality. Each criterion was indicated as met (Y) if it fully met the above criteria, not met (N) if it did not, or uncertain (U) if the criteria were not mentioned in the text, if the report was unclear, or if there was no response from the authors after multiple email contacts.

### Data Analysis

A meta-analysis was conducted to explore the effects of qigong and tai chi on addiction. The pre- and post-intervention measurements of the experimental and control groups were entered into the Comprehensive Meta-Analysis (CMA) V2, and the results of the total effects from random effects (overall) were selected to evaluate the effects of qigong and tai chi on addiction. In addition, mixed effects analysis results were selected to evaluate the differences of between groups (qigong *vs*. tai chi). The results of the data analysis are subject to the possibility of heterogeneity due to differences in the intervention period, the characteristics of the participants, and the scales used in the included studies. To account for potential heterogeneity, random effect (standardized mean difference [SMD], 95% CI) models were used throughout the data synthesis and analysis process. *I*^2^ = 0 indicated no heterogeneity among studies; *I*^2^ = 25–50% indicated low heterogeneity among studies; *I*^2^ = 50–75% indicated moderate heterogeneity among studies; and I^2^ > 75% indicated high heterogeneity among studies (54).

## Results

### Literature Screening

Figure 1 summarizes the process and results of the literature screening. In total, 2,667 articles were retrieved by the search method mentioned above, 168 duplicates were excluded, and title screening (*n* = 2,499) was then performed to exclude 2,364 articles that did not meet the criteria. Subsequently, the remaining articles (*n* = 135) were screened for abstracts, and 102 articles that did not meet the criteria were excluded. The remaining articles (*n* = 33) were then screened in full to exclude 20 studies that did not meet the criteria, resulting in the inclusion of 13 studies that met the criteria (randomized controlled trials = 12, quasi-experiments = 1). Kappa (K) coefficients were calculated for the literature that was screened independently by the two researchers, and the results showed that the Kappa coefficients for both the abstract screening (*K* = 0.85) and full-text screening (*K* = 0.84) were > 0.75, indicating that the degree of consistency between the two researchers' literature screening results was good.

### Characteristics of Included Studies

Table 1 shows the characteristics of the included studies. The included studies five (15, 43, 44, 49, 55) in English and six (41, 42, 45–48) in Chinese were all published in peer-reviewed journals. Of these, two studies (46, 47) were RCTs and nine (15, 41, 42, 44–48, 55) were NRSs. Ten studies were conducted in China (15, 41–48, 55), and one study did not mention the location of the trial in the text (43). The drug addicted participants included heroin, methamphetamine, amphetamine-type stimulants, and synthetic drug dependents, with sample sizes ranging from 59 to 200, for a total of 1,072 subjects (546 in the intervention group and 527 in the control group); six studies (15, 41, 42, 44, 48, 55) had all female participants, and one study (49) recruited all male participants.

The interventions in the experimental group were qigong exercises (e.g., Wu Qin Xi, Ba Duan Jin, Dao Yin, Pan Gu qigong) or tai chi (e.g., 24-form tai chi). The interventions in the control group were treatment as usual, medication, daily physical exercise, and no treatment. All studies considered only the immediate effects and impact of qigong exercise in drug addicts, with few studies providing follow-up measurements. In terms of outcome indicator measures, five studies (15, 41–44) measured depression variables by self-reported questionnaires (e.g., HRSD, BDI, SCL-90, SDS), and five studies (41–43, 45, 55) measured anxiety variables by various questionnaires (e.g., SAMA, STAI, SAS, SCL-90). Three studies (47–49) focused on the quality of life (e.g., QOL-DA), two studies (41, 44) used subjectively-measured sleep quality (e.g., PSQI).

### Quality Evaluation

Table 2 shows the results of the quality evaluation of the studies included. Only two studies (18%) (43, 44) used a random number table or computer-generated random numbers for random assignment of subjects. Only two studies (18%) (42, 48) implemented allocation concealment. Ten studies (91%) (15, 41–45, 47–49, 55) provided details of each randomized grouping intervention. Only four studies (36%) (42, 43, 47, 48) had details on the experience or skills of the person implementing the intervention that were appropriate. Only one study (9%) (Li, 2018) provided a quantitative assessment of subject (e.g., patients) compliance. Nine studies (15, 41–47, 49, 55) showed no significant difference in the number of subjects who dropped out and were lost to follow-up in the experimental and control groups. Similarly, although only one study (9%) (43) implemented adequate blinding of intervenors, nine studies (15, 42–49) had intervenors who provided consistent alternative treatment and care for each randomized subgroup. Six studies (86%) (15, 42–44, 48, 49) were adequately blinded to those assessing the outcome variable. Nine studies (82%) (15, 41–45, 47–49) implemented fully parallel intervention plans in the experimental and control groups. Looking at the individual studies, the overall quality was poor, with only one study (43) classified as good quality, meeting 83% of the evaluation criteria; two studies (42, 47) were of fair quality, meeting 64 and 69% of the evaluation criteria respectively; eight studies [(15, 44, 48, 49, 55); Huang and Xu, 2015; Fu, 2016; Huang and Wu, 2017] were of substandard quality, in which only 33, 50, 46, 50, 45, 50, 33, and 57% of the evaluation criteria were met, respectively.

**Table 2:** Critical appraisal of included studies.

**Criteria**	**Li et al. (15)**	**Smelson et al. (43)**	**Zhu et al. (44)**	**Geng et al. (42)**	**Fu et al. (41)**	**Huang et al. (45)**	**Li et al. (47)**	**Zhu et al. (48)**	**Zhu et al. (49)**	**Huang et al. (46)**	**Li et al. (55)**	**Score^a^ (% Y)**
1. Was the generation of allocation adequate?	U	Y	Y	N	U	U	U	N	N	U	U	18%
2. Was the treatment allocation concealed?	U	U	N	Y	U	U	N	Y	N	U	U	18%
3. Were details of the intervention administered to each group made available?	Y	Y	Y	Y	Y	Y	Y	Y	Y	U	Y	91%
4. Were care providers' experience or skills in each arm appropriate?	U	Y	U	Y	U	U	Y	Y	U	U	U	36%
5. Was participant (i.e., patients) adherence assessed quantitatively?	U	U	U	N	U	U	Y	N	U	U	U	9%
6. Were participants (i.e., patients) adequately blinded? if no, go to point 6.1 and 6.2	N	Y	N	N	N	N	N	N	N	N	N	9%
6.1. Were other treatments and care (i.e. co-interventions) the same in each randomized group?	Y	N/A	Y	Y	N/A	Y	Y	Y	Y	Y	U	89%
6.2. Were withdrawals and lost to follow up the same in each randomized group?	Y	N/A	Y	Y	Y	Y	Y	N	Y	Y	Y	90%
7. Were care providers for the participants adequately blinded? if no, go to point 7.1 and 7.2	U	Y	U	N	N	N	N	N	U	N	U	9%
7.1. Were other treatments and care (i.e. co-interventions) the same in each randomized group?	Y	Y	Y	Y	N/A	Y	Y	Y	Y	Y	N/A	100%
7.2. Were withdrawals and lost-to-follow-up the same in each randomized group?	Y	Y	Y	Y	Y	Y	Y	N	Y	Y	Y	91%
8. Were outcome assessors adequately blinded to assess the primary outcomes? If no, go to 8.1	Y	Y	Y	Y	N/A	N/A	N/A	Y	Y	U	N/A	86%
8.1. If outcome assessors were not adequately blinded, were specific methods used to avoid ascertainment bias?	N/A	N/A	N/A	N/A	N/A	N/A	N/A	N/A	N/A	U	N/A	0%
9. Was the follow-up schedule the same in each group? (parallel design)	Y	Y	Y	Y	Y	Y	Y	Y	Y	U	U	82%
10. Were the main outcomes analyzed according to the intention-to-treat principle?	N	Y	N	N	Y	N	Y	N	N	Y	Y	45%
Score^b^ (% Y)	50%	83%	57%	64%	45%	46%	69%	50%	50%	33%	33%	

*Y, yes; N, no; N/A, not appropriate (not included in the score); U, unable to determine; Score^a^ (% Y), percentage of the number of studies scoring “Yes” for each criterion*;

*Score^b^ (% Y), percentage of the number of “Yes” scoring for each study*.

### Effects of Qigong and Tai Chi on Depressive Symptoms

**Figure 2:**
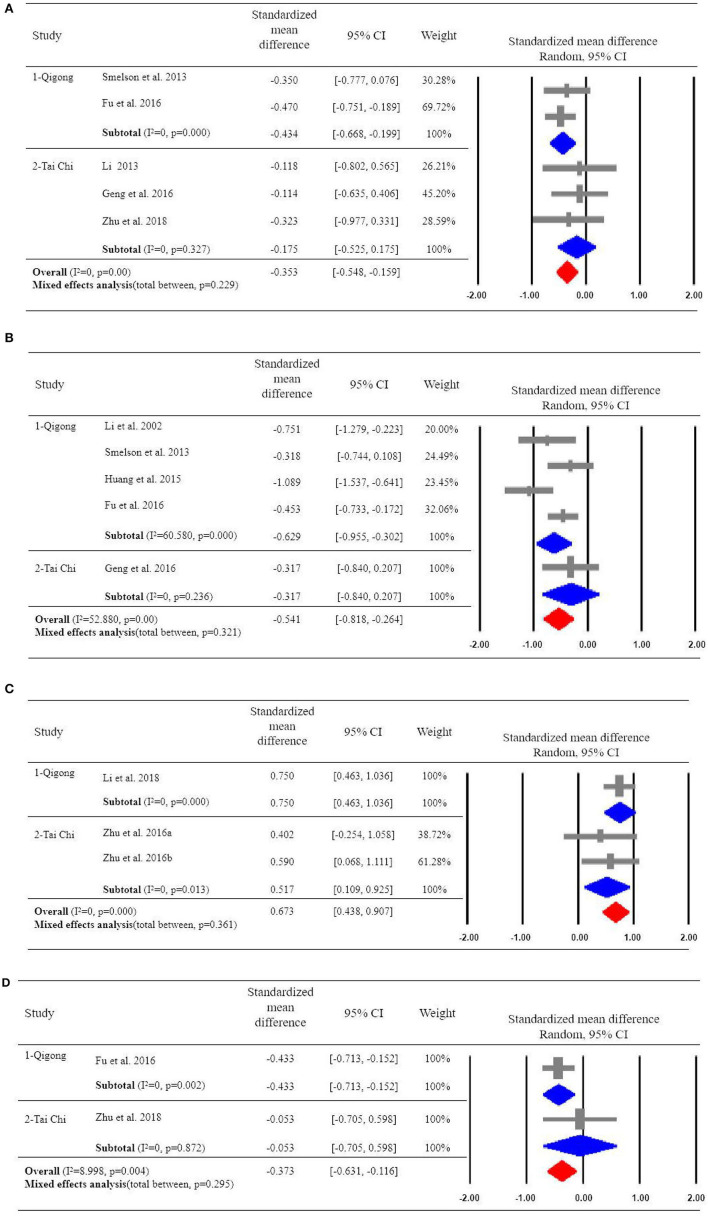
**(A)** Effects of Qigong or Tai Chi on depressive symptoms. **(B)** Effects of Qigong or Tai Chi on anxiety symptoms. **(C)** Effects of Qigong or Tai Chi on Quality of life scale for drug addicts. **(D)** Effects of Qigong or Tai Chi on Pittsburg Sleep Quality Index.

Five of the included studies examined the effects of qigong or tai chi exercise on depression (15, 41–44). Overall, qigong and tai chi had a significant effect on alleviating depression in patients with drug addiction (SMD = −0.353, 95%CI [−0.548, −0.159], *p* = 0.000), with high homogeneity (*I*^2^ = 0). We also implemented the subgroup analyses to compare the effects of qigong and tai chi on depressive symptoms, indicating a significant effect of qigong on depression (SMD = −0.434, 95%CI [−0.668, −0.199]) with high homogeneity (*I*^2^ = 0); but non-significant effect of tai chi exercise on the improvement of depressive symptoms (SMD = −0.175, 95%CI [−0.525, 0.175]) (Figure 2A).

### Effects of Qigong and Tai Chi on Anxiety Symptoms

Five of the included studies examined the effects of qigong or tai chi exercise on anxiety symptoms (41–43, 45, 55). In total, qigong and tai chi exercise had a significant overall effect on alleviating anxiety symptoms in patients with drug addiction (SMD = −0.541, 95%CI [−0.818, −0.264], *p* = 0.000), with moderate heterogeneity (*I*^2^ = 52.88). The subgroup analysis showed that qigong exercise had a significant effect on decreasing anxiety (SMD = −0.629, 95%CI [−0.955, −0.302]) with high heterogeneity (*I*^2^ = 60.58). However, tai chi exercise reported a non-significant effect on anxiety (SMD = −0.317, 95% CI [−0.840, 0.207]) with high homogeneity (*I*^2^ = 0) (Figure 2B).

### Impact of Qigong and Tai Chi on Quality of Life

Three of the included studies examined the effect of qigong or tai chi exercise on quality of life (47–49). The overall effects showed that qigong and tai chi had a significant effect on improving the quality of life of patients with drug addiction (SMD = 0.673, 95%CI [0.438, 0.907]), with high homogeneity (*I*^2^ = 0). Among the studies, one study (47) examined the effect of qigong exercise on quality of life in drug addicts (SMD = 0.750, 95%CI [0.463, 1.036]). Two studies (48, 49) examined the effect of tai chi exercise on the quality of life of drug addicts (SMD = 0.517, 95%CI [0.109, 0.925]), with high homogeneity (*I*^2^ = 0) (Figure 2C).

### Effects of Qigong and Tai Chi on Sleep Quality

Two of the included studies examined the effect of qigong or tai chi exercise on sleep quality (PSQI) (41, 44). The results showed that qigong and tai chi exercise had a significant overall effect on relieving anxiety symptoms in patients with drug addiction (SMD = −0.373, 95%CI [−0.631, −0.116]), with low heterogeneity (*I*^2^ = 8.998) (Figure 2D).

## Discussion

This review summarizes the effects of qigong and tai chi exercise on patients with drug addiction. Overall, qigong and tai chi exercise can improve depression, anxiety, quality of life, and sleep quality. The subgroup analyses showed that qigong exercise outperformed tai chi on alleviating addiction-related symptoms.

We found that qigong and tai chi exercise produced a significant effect on improvement of depression and anxiety in patients with drug addiction. It is probably because improvements in general physical health or reductions in chronic disease symptoms may contribute to improvements in patients' mental health. Although subgroup analysis found that qigong is significantly associated with improved anxiety symptoms, there exists high heterogeneity among the included of studies (*I*^2^ = 60.580). This may be due to the different measurement tools and interventions used in the study, as well as the intervention periods. However, in the subgroup analysis of tai chi, the effects of tai chi exercise on patient depression and anxiety were not significant, which may be related to the following aspects. First, compared to tai chi, qigong may focus more on the flow of “qi” (e.g., breath and intention) in the body and the effects from it. According to Chinese medical philosophy, qigong believes that the human body is a “small universe” and that diseases occur because the circulation of “qi” (vital energy) in the human body is blocked. Qigong is considered to be a method to achieve the harmonious flow of vital energy in the human body and to regulate the functional activities of the meridians and internal organs (22, 33, 34, 56–58). Qigong exercises always require the practitioner to integrate the body, breath, and the mind (spirit), whether it be dynamic or static qigong, and consciously guiding the flow of “qi” in the body is the most crucial and core element of the practice (59, 60). Secondly, qigong has a much longer history than tai chi in promoting the development of human mental health. “Promoting healthy development of the body” has always been the unchanging function and principle of qigong. In contrast, tai chi originally existed as a martial art, consisting of movements with offensive and defensive functions. Only with cultural changes did it evolve into many gentler styles, and its function or intention gradually tilted toward mental health promotion (59). Thirdly, the small number of studies available on tai chi exercise (four papers on qigong exercise, but only one on tai chi exercise. Finally, from the available evidence and the overall results of the two qigong and tai chi subgroups, it appears that qigong and tai chi exercises are potentially beneficial in improving patients' depression and anxiety symptoms, but more rigorous studies are needed in the future to verify this.

Compared to depression and anxiety, less research focused on quality of life in drug addiction patients. Quality of life reflects the patient's perception of personal health and life satisfaction over time (61). Research in this area suggests that addiction can affect various aspects of daily functioning, including physical health (62, 63), and social functioning (64). Individuals with drug addiction tend to have a lower level of quality of life compared to the general population (65–67). Based on our findings, both qigong and tai chi interventions showed significant improvement in the quality of life of drug addiction patients. Specifically, patients showed significant improvements in somatic functioning, psychological functioning, social functioning, and symptoms/side effects after qigong or tai chi interventions, which are consistent with the results of previous studies (68, 69).

Compared to the outcome variables mentioned above, from the studies included in this paper, it is evident that researchers have paid less attention to sleep quality, heroin dependence protracted withdrawal symptoms, and cocaine craving symptoms. However, a large body of research (43, 70–74) shows that these three factors are also very important indicators for drug addiction patients, and there are reviews that have confirmed qigong or tai chi interventions to be effective as well (6, 15, 43, 75). Although our study also found similar significant effects, the number of articles was too small to classify our results as robust. More attention and research could be devoted to this research in the future, as the improvement of different symptoms is always beneficial for the physical and mental health of drug addiction patients.

This review has several potential limitations. First, the quality included studies was relatively low (e.g., two RCTs), making it difficult to judge the effectiveness and provide clear conclusions about qigong and tai chi interventions. Second, only 11 articles were included in the meta-analysis, making it difficult to have reliable results. Third, some of the patients included in the articles were provided a combination of medication and qigong and tai chi interventions, making it difficult for us to determine whether the results were due to the efficacy of qigong itself or the synergistic effect of the medications and the exercises. Fourth, the intervention designs, selection processes, and measurements of outcome variables varied considerably, which could lead to some uncertainty in our results. Last but not the least, subgroup analysis for sex comparison was not allowed in this meta-analysis due to large gender inequality of these included studies as well as the absence of independent results across gender in those trials with both males and females.

## Conclusion

This meta-analysis suggests that qigong and tai chi can be effective in improving depression, anxiety, and quality of life in patients with drug addiction, validating their use as adjunctive treatments for depression, anxiety, and quality of life in drug users. However, the effectiveness of qigong and tai chi in improving sleep quality, heroin dependence protracted withdrawal symptoms; cocaine craving in these patients is unclear due to the paucity of the literature, and more studies in this area are needed in the future to validate their efficacy and effects.

## Data Availability Statement

The original contributions presented in the study are included in the article/supplementary material, further inquiries can be directed to the corresponding author.

## Author Contributions

JC: conceptualization, formal analysis, and writing—original draft. FL: screening. XL: screening and formal analysis. RL: formal analysis and writing—review and editing. XC and HZ: writing—review and editing. All authors contributed to the article and approved the submitted version.

## Funding

This work was partially supported by a grant from the National Social Science Fund of China (Grant no. 13&ZD140).

## Conflict of Interest

The authors declare that the research was conducted in the absence of any commercial or financial relationships that could be construed as a potential conflict of interest.

## Publisher's Note

All claims expressed in this article are solely those of the authors and do not necessarily represent those of their affiliated organizations, or those of the publisher, the editors and the reviewers. Any product that may be evaluated in this article, or claim that may be made by its manufacturer, is not guaranteed or endorsed by the publisher.
